# Chemoptogenetic damage to mitochondria causes rapid telomere dysfunction

**DOI:** 10.1073/pnas.1910574116

**Published:** 2019-08-26

**Authors:** Wei Qian, Namrata Kumar, Vera Roginskaya, Elise Fouquerel, Patricia L. Opresko, Sruti Shiva, Simon C. Watkins, Dmytro Kolodieznyi, Marcel P. Bruchez, Bennett Van Houten

**Affiliations:** ^a^Department of Pharmacology and Chemical Biology, University of Pittsburgh School of Medicine, Pittsburgh, PA 15213;; ^b^University of Pittsburgh Medical Center (UPMC) Hillman Cancer Center, University of Pittsburgh School of Medicine, Pittsburgh, PA 15213;; ^c^Molecular Genetics and Developmental Biology Graduate Program, University of Pittsburgh School of Medicine, Pittsburgh, PA 15213;; ^d^Department of Environmental and Occupational Health, University of Pittsburgh Graduate School of Public Health, Pittsburgh, PA 15261;; ^e^Vascular Medicine Institute, University of Pittsburgh School of Medicine, Pittsburgh, PA 15261;; ^f^Department of Cell Biology and Physiology, University of Pittsburgh, Pittsburgh, PA 15261;; ^g^Center for Biological Imaging, University of Pittsburgh, Pittsburgh, PA 15261;; ^h^Department of Chemistry, Molecular Biosensors and Imaging Center, Carnegie Mellon University, Pittsburgh, PA 15213;; ^i^Department of Biological Sciences, and Molecular Biosensors and Imaging Center, Carnegie Mellon University, Pittsburgh, PA 15213

**Keywords:** ATM signaling, DNA damage response, singlet oxygen, mitochondria, telomere

## Abstract

Reactive oxygen species (ROS) play important roles in aging, inflammation, and cancer. Mitochondria are an important source of ROS; however, the spatiotemporal ROS events underlying oxidative cellular damage from dysfunctional mitochondria remain unresolved. To this end, we have developed and validated a chemoptogenetic approach that uses a mitochondrially targeted fluorogen-activating peptide (Mito-FAP) to deliver a photosensitizer MG-2I dye exclusively to this organelle. Light-mediated activation (660 nm) of the Mito-FAP–MG-2I complex led to a rapid loss of mitochondrial respiration, decreased electron transport chain complex activity, and mitochondrial fragmentation. Importantly, one round of singlet oxygen produced a persistent secondary wave of mitochondrial superoxide and hydrogen peroxide lasting for over 48 h after the initial insult. By following ROS intermediates, we were able to detect hydrogen peroxide in the nucleus through ratiometric analysis of the oxidation of nuclear cysteine residues. Despite mitochondrial DNA (mtDNA) damage and nuclear oxidative stress induced by dysfunctional mitochondria, there was a lack of gross nuclear DNA strand breaks and apoptosis. Targeted telomere analysis revealed fragile telomeres and telomere loss as well as 53BP1-positive telomere dysfunction-induced foci (TIFs), indicating that DNA double-strand breaks occurred exclusively in telomeres as a direct consequence of mitochondrial dysfunction. These telomere defects activated ataxia-telangiectasia mutated (ATM)-mediated DNA damage repair signaling. Furthermore, ATM inhibition exacerbated the Mito-FAP–induced mitochondrial dysfunction and sensitized cells to apoptotic cell death. This profound sensitivity of telomeres through hydrogen peroxide induced by dysregulated mitochondria reveals a crucial mechanism of telomere–mitochondria communication underlying the pathophysiological role of mitochondrial ROS in human diseases.

## Significance

It is highly controversial whether secondary reactive oxygen species generated by dysfunctional mitochondria are able to diffuse across the cytoplasm to the nucleus and cause subsequent nuclear changes. We have developed a targeted chemoptogenetic technology to induce mitochondrial dysfunction by generating short-lived highly reactive singlet oxygen exclusively in the mitochondria, with precise spatiotemporal control by light stimulation. Through careful analysis of the events involving mitochondrial dysfunction and subsequent nuclear oxidative stress that resulted in specific telomere damage, we delineated the mechanism of mitochondria–telomere axis of cellular damage. Our findings revealed a fundamental mechanism underlying the pathophysiological role of mitochondrial singlet oxygen, with important ramifications for understanding the role of mitochondrial signaling in aging and cancer among other human diseases.

Reactive oxygen species (ROS) are produced in biological systems through both intrinsic and extrinsic factors, including mitochondrial electron transport, inflammation, and environmental stressors (1). Mitochondria are an important source and also, the targets of ROS (2). Dysfunctional mitochondria are associated with a wide range of human maladies, including aging, cancer, cardiovascular disease, ischemia reperfusion injury, and neurodegeneration (3, 4). However, the precise role of mitochondrial-produced ROS and its temporal events in pathophysiological conditions, such as aging and cancer, is not clear. ROS damage to mitochondria can initiate a continuous cycle of ROS generation including superoxide and hydrogen peroxide (5), which further amplifies oxidative damage to mitochondrial proteins and DNA, leading to cellular functional alterations, such as metabolism and survival (6). Although ROS is believed to play a role in mitochondria–nuclear anterograde and retrograde signaling (7), the question as to whether ROS generated secondary to mitochondrial dysfunction are able to directly reach to the nucleus and cause direct nuclear DNA damage is highly controversial and remains unresolved. For instance, it has been reported that intrinsic mitochondrial ROS, when stimulated by electron transport chain (ETC) complex I inhibitor rotenone or inactivating mutation of Cockayne syndrome B, is not able to cause oxidative damage to nuclear DNA in human fibroblasts in vitro (8). Other than the use of ETC inhibitors to directly stimulate ROS, glutathione depletion to suppress ROS elimination also did not cause oxidative base modifications in the nuclear DNA (9). However, chronic mitochondrial ROS stress in mice partially deficient in manganese superoxide dismutase (MnSOD) has been shown to elevate levels of 8-oxo-2-deoxyguanosine in nuclear DNA later in life (10). Furthermore, mitochondria-targeted DNA oxidizing agents have been shown to only be able to induce mitochondrial-specific oxidative stress in the absence of cytoplasm and nuclear ROS accumulation (11). Among nuclear alterations, shortening of telomeres has been considered as a consequence of oxidative stress, although the role of mitochondria in oxidative telomere damage is not clear (12). Similarly, telomere damage as indicated by telomere dysfunction-induced foci (TIFs) has been observed in senescent cells with higher mitochondrial superoxide generation; however, the cause and effect relationship between mitochondrial ROS and telomere damage has not yet been established (13, 14).

To directly address the cellular consequences of mitochondrial dysfunction and subsequent ROS production, it is necessary to develop a highly targeted approach that can be tightly regulated to produce direct mitochondrial damage without spurious damage to other cellular components. One promising approach is to use chromophore-assisted light inactivation technology that combines light activation with a fluorescent moiety to produce ROS, particularly with newer genetically encoded photosensitizer proteins, such as KillerRed or MiniSOG (mini singlet oxygen generator) (15, 16). Genetic targeting of these fluorescent moieties to specific cellular compartments by fusion to organelle-specific targeting peptides has provided high spatial delivery. However, problems of spurious light activation of these intrinsic photosensitizer proteins or other intrinsic biological photosensitizers (e.g., flavins) persist (17). An ideal solution would combine light in the near-infrared (NIR) range, avoiding intrinsic chromophore activation, with an organelle-targeted fluorescent protein that is only activated by the addition of a high-affinity chemical ligand, avoiding issues related to continuous excitation of the photosensitizer by ambient light.

To this end, we have developed and validated a unique chemoptogenetic approach consisting of a binding-activated photosensitizer dye MG-2I (iodine-substituted malachite green analog) and a mitochondrial-targeted fluorogen-activating peptide (Mito-FAP) to produce on-demand, short-lived singlet oxygen in the mitochondrial matrix (18, 19). Unbound MG-2I has a low fluorescence and undetectable singlet oxygen generation; however, when bound to Mito-FAP and exposed to NIR light (660 nm), the resulting complex is highly fluorescent and produces singlet oxygen with a high quantum yield. This system provides precise spatiotemporal control of generation of singlet oxygen to only the mitochondria. Singlet oxygen is formed by the transfer of energy to ground-state triplet molecular oxygen through various enzymatic and nonenzymatic systems, such as processes mediated by photosensitizers, peroxidase enzymes, and radical termination reactions (20). Singlet oxygen is highly reactive and therefore, short lived (2 to 4 μs), attacking a wide range of biological targets, including DNA, RNA, proteins, and lipids only in close proximity to the site of generation. Singlet oxygen directly oxidizes amino acid side chains of proteins, including His, Tyr, Met, Cys, and Trp, at physiological pH, causing loss of enzyme activities (20). Using the Mito-FAP system, we have directly addressed whether dysfunctional mitochondria are able to generate sufficient fluxes of ROS to attack other cellular compartments, including nuclear DNA. Our study revealed that damage to mitochondria inflicted by mitochondrial-targeted singlet oxygen produced a long-lived wave of secondary ROS, which caused mtDNA damage, ATM activation, cell cycle arrest, and rapid telomere dysfunction as characterized by 53BP1-positive TIFs, telomere fragility, and telomere loss in the apparent absence of general nuclear DNA strand breaks. Our finding provided direct evidence that mitochondrial dysfunction has profound effects on the nucleus, with important ramifications for the role of mitochondrial-generated ROS in aging and cancer.

## Results

### Mito-FAP System Induces Mitochondrial Dysfunction.

**Fig. 1.**
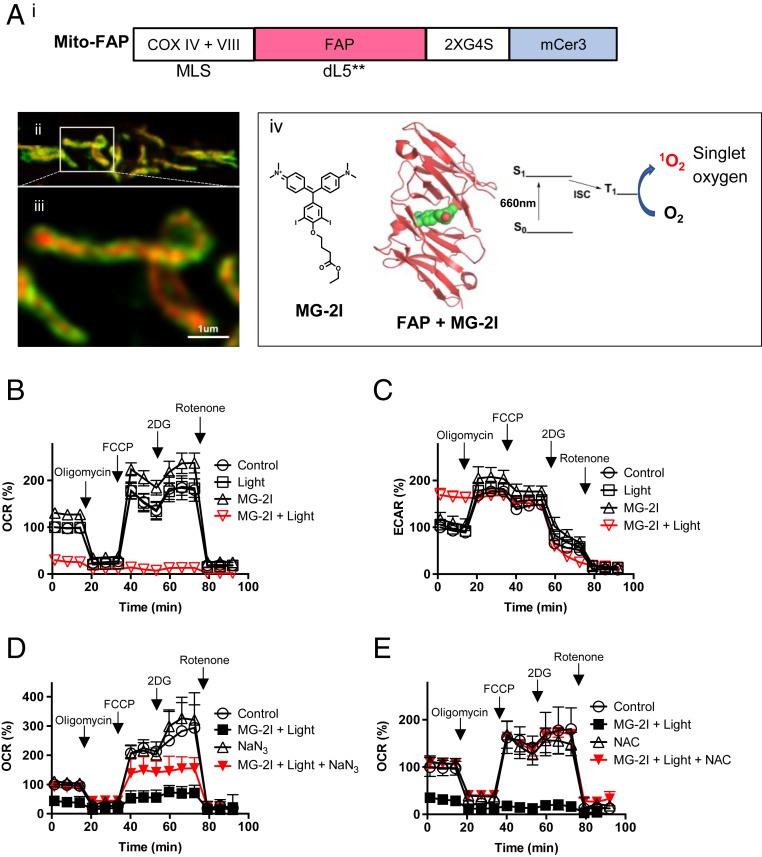
ROS exclusively generated in mitochondria by a Mito-FAP system induces mitochondrial dysfunction. (*A*) The structure of the Mito-FAP plasmid construct (*i*), STED images of localization of Mito-FAP (*ii* and *iii*), and the mechanism of generation of singlet oxygen on binding of MG-2I with FAP after light exposure (*iv*). *A*, *ii* shows a low-magnification single-plane STED image of HEK293 cells expressing mNEON-TOM20 (green) and Mito-FAP (red; imaged through binding to MG-ester). The red signal of Mito-FAP is clearly contained within the green mNEON profile. This is more clearly shown in the enlargement (*A*, *iii*). (*B* and *C*) HEK293 cells stably expressing Mito-FAP were treated with MG-2I dye (50 nM) alone, light exposure (660 nm, 5 min) alone, or light exposure (660 nm, 5 min) in the presence of 50 nM MG-2I. Mitochondrial respiration as determined by OCR and ECAR was assessed by a Seahorse Extracellular Flux Analyzer 4 h after treatment. (*D* and *E*) HEK293 Mito-FAP cells were treated with or without a singlet oxygen scavenger sodium azide (50 mM; *D*) or a broad-spectrum ROS scavenger NAC (10 mM; *E*) added 15 min before exposure to MG-2I dye (50 nM) and light (5 min). Four hours after treatment, OCR was determined by a Seahorse Extracellular Flux Analyzer. Data show the representative results of at least 3 experiments with similar results. Data are represented as mean ± SD of at least 6 wells. MLS, mitochondrial leading sequence.

We used HEK293 cells stably expressing Mito-FAP (18, 19) to investigate the cellular impact of singlet oxygen-induced mitochondrial dysfunction (Fig. 1*A*). This chemoptogenetic approach consists of an inert fluorogen-activating peptide (FAP) targeted to the mitochondrial matrix (Fig. 1 *A*, *ii* and *iii*) with mitochondrial leading sequences of COXIV and COXVIII (Fig. 1 *A*, *i*) combined with an MG-2I dye that is activated by NIR light, producing singlet oxygen when bound by the targeted FAP (Fig. 1 *A*, *iv*) (18, 19). As shown in Fig. 1*B*, 4 h after dye MG-2I (50 nM) and light exposure (660 nm, 5 min), we observed a reduction in basal oxygen consumption rate (OCR) of cells as well as a diminished response to mitochondrial uncoupler carbonyl cyanide 4-(trifluoromethoxy)phenylhydrazone (FCCP) compared with MG-2I dye or light treatment alone. In comparison, we did not observe any effect of MG-2I and light on cells that do not express Mito-FAP (*SI Appendix*, Fig. S1), confirming the specificity of the Mito-FAP chemoptogenetic system. The decrease in OCR is also dependent on the duration of light exposure (*SI Appendix*, Fig. S2). Correspondingly, there was a compensatory increase of extracellular acidification rate (ECAR) in MG-2I– and light-treated cells (Fig. 1*C*). Sodium azide, a singlet oxygen quencher, when present during light activation was able to rescue basal oxygen consumption and partially restore the response to FCCP after MG-2I and light treatment (Fig. 1*D*), indicating that mitochondrial dysfunction induced by the Mito-FAP system is in part mediated by singlet oxygen. *N*-acetyl-cysteine (NAC), a scavenger of a wide range of free radicals, including both singlet oxygen and other ROS (21, 22), when present during and after light exposure provided a nearly complete protection of mitochondrial respiration (Fig. 1*E*) (both basal and response to FCCP), suggesting that ROS other than singlet oxygen alone are involved in the mitochondrial dysfunction observed 4 h after MG-2I and light treatment. Taken together, mitochondrial-targeted generation of singlet oxygen leads to a drastic decline of mitochondrial function, which can be mitigated by either singlet oxygen quenching or a broad-spectrum antioxidant.

### Mitochondrial Singlet Oxygen Induces Delayed Decrease of ETC Activities.

**Fig. 2.**
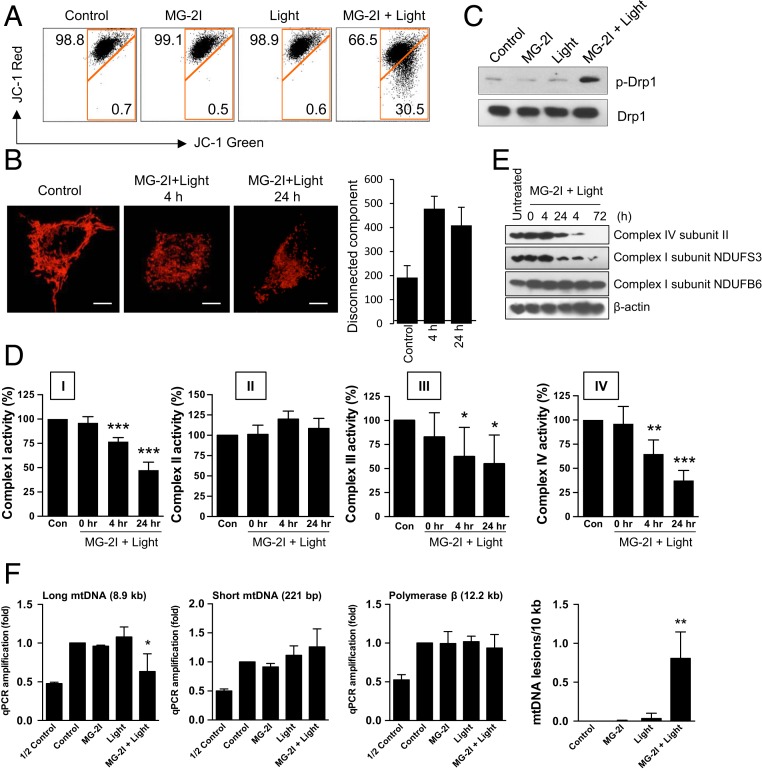
Characterization of mitochondrial damage induced by mitochondrial singlet oxygen generation through Mito-FAP. (*A*) HEK293 Mito-FAP cells were treated with MG-2I dye (50 nM) alone, light exposure (660 nm, 5 min) alone, or light exposure (660 nm, 5 min) in the presence of 50 nM MG-2I. The mitochondrial membrane potential was assessed by a membrane potential indicator JC-1 24 h after treatment. The loss of JC-1 red fluorescence after MG-2I and light treatment indicates the shift from JC-1 dimer to JC-1 monomer and hence, the loss of mitochondrial membrane potential. (*B*) At 4 and 24 h after treatment with 50 nM MG-2I and light (660 nm, 5 min), mitochondrial morphology was visualized by staining cells with Tom20 and Alexa 555-conjugated secondary antibody. Mitochondrial connectivity was quantified by reconstructed 3-dimensional images using Imaris software. (Scale bars: 5 μm.) (*C*) Phosphorylation of Drp1 was determined by western blot 24 h after treatment as indicated. (*D*) HEK293 Mito-FAP cells were treated with or without the combination of MG-2I (50 nM) and light (5 min). For the determination of ETC complex activities, cells were harvested right after treatment (0 h) or after 4 and 24 h of recovery. Data represent mean ± SEM of at least 3 independent experiments. *P < 0.05; **P < 0.01; ***P < 0.001. (*E*) Changes in the protein levels of several subunits of ETC complexes were evaluated by western blot at the indicated time points after MG-2I (50 nM) and light (5-min) treatment. (*F*) HEK293 Mito-FAP cells were treated with MG-2I dye (50 nM) alone, light exposure (660 nm, 5 min) alone, or light exposure (660 nm, 5 min) in the presence of 50 nM MG-2I. The damage of mtDNA and nuclear DNA was analyzed by a long-range qPCR DNA lesion assay 24 h after treatment. The PCR amplification of a large mtDNA segment (8.9 kb), a small mtDNA segment (221 bp), and the gene of polymerase β (12.2 kb) was performed. The inclusion of the measurement of amplification with a template DNA amount that is half of the amount used in control group indicates that the amount of PCR product corresponds to the starting amount of template DNA. Data represent mean ± SEM of 3 independent experiments with 3 PCR reactions per treatment condition. *P < 0.05 (one-way ANOVA); **P < 0.01 (one-way ANOVA).

Singlet oxygen damage to mitochondria after photodynamic therapy has been suggested to cause lipid damage, rapid loss of inner mitochondrial membrane potential, subsequent cytochrome *c* release, and cell death (23). Using a monovalent cationic fluorescent probe tetraethylbenzimidazolylcarbocyanine iodide (JC-1), we observed that 30.5% of cells underwent red-to-green shift of JC-1 fluorescence 24 h after MG-2I and light treatment, indicating a significant loss of mitochondrial membrane potential after controlled singlet oxygen generation by the Mito-FAP system (Fig. 2*A*). Quantification of mitochondrial connectivity revealed more disconnected mitochondria in a time-dependent manner after MG-2I and light treatment, indicating mitochondrial fragmentation after singlet oxygen-initiated oxidative stress (Fig. 2*B*). The observation of fragmentation of mitochondria was also supported by the finding of phosphorylation of a mitochondrial fission protein Drp1 24 h after treatment (Fig. 2*C*). In addition, we also observed a significant decrease in the activities of mitochondrial ETC complexes I (47.4% of control), III (65.3% of control), and IV (37.5% of control) (Fig. 2*D*) but not complex II 24 h after MG-2I and light treatment. The decrease in the activities of ETC complexes starting from as fast as 4 h is consistent with the decrease of OCR at 4 h shown in Fig. 1*B*. Furthermore, we observed a decrease in the protein levels of certain ETC subunits (e.g., Complex IV subunit II and Complex I subunit NDUFS3) (Fig. 2*E*). The prolonged mitochondrial response after completion of light exposure suggested that the continuous damage occurring after the initial burst of singlet oxygen generation during light exposure is probably mediated by downstream ROS intermediates from compromised ETC. Furthermore, using a qPCR-based mtDNA damage assay (24), we observed a reduced amplification of a long fragment (8.9 kb) of mtDNA at 24 h after MG-2I plus light treatment, which is equivalent to an average 0.8 lesions per 10 kb of mtDNA (Fig. 2*F*). In contrast, the amplification of nuclear gene of polymerase β (12.2 kb) was not affected, indicating mitochondria-specific oxidative DNA damage by the Mito-FAP system.

### Mitochondrial Singlet Oxygen Triggers a Secondary Wave Generation of Superoxide and Hydrogen Peroxide.

**Fig. 3.**
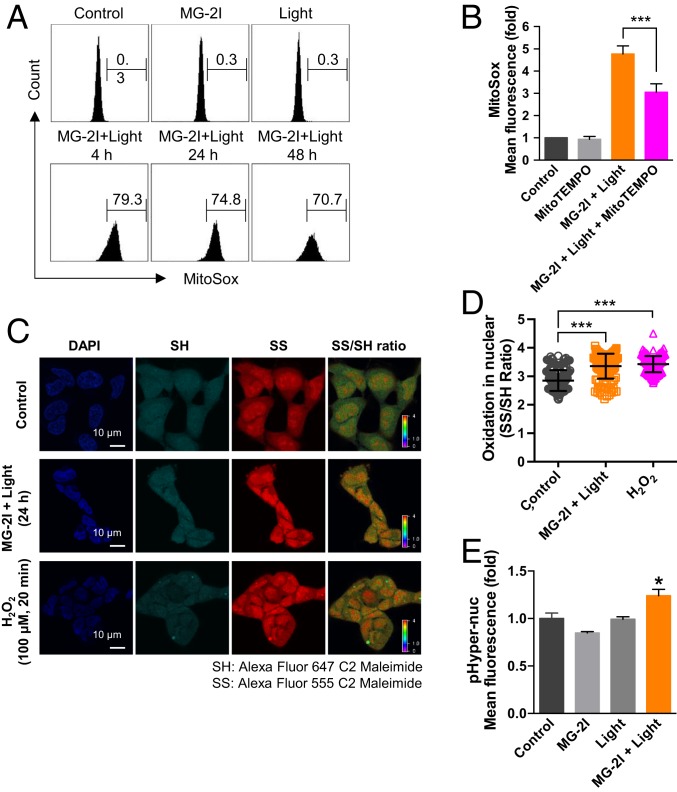
Singlet oxygen-induced mitochondrial dysfunction leads to a secondary wave generation of ROS. (*A*) Mitochondrial generation of ROS was determined by MitoSox at 4, 24, and 48 h after treatment of HEK293 Mito-FAP cells with MG-2I (50 nM), light (5 min), or light (5 min) in the presence of MG-2I (50 nM). (*B*) MitoTEMPO (100 nM) inhibited mitochondrial superoxide generation 4 h after treatment. ***P < 0.001. (*C*) Oxidation of protein SH group was evaluated by thiol oxidation immunocytochemistry 24 h after treatment with light (5 min) in the presence of MG-2I (50 nM). Treatment of 100 μM H_2_O_2_ for 20 min served as the positive control. Representative images are shown. (*D*) Quantification on the ratio of fluorescence intensity of Alexa Fluor 555/647 (SS/SH) in the nuclear region of the cells. Data are represented as mean ± SD. ***P < 0.001. (*E*) Detection of diffusion of H_2_O_2_ into nuclei by a fluorescent nuclear H_2_O_2_ sensor pHyper-nuc 24 h after treatment with light (5 min) in the presence of MG-2I (50 nM). Data are represented as mean ± SEM. *P < 0.05.

The duration of singlet oxygen generation in mitochondria by the mitochondrial-targeted Mito-FAP system can be precisely controlled by the time of exposure to light, which in our study, is 5 min. The lifetime of singlet oxygen in most solvents is in the microsecond range (25). Since we did not detect immediate damaging effects of singlet oxygen on mitochondrial function (Fig. 2) and because NAC had a higher protective effect against MG-2I and light-induced mitochondrial dysfunction than sodium azide, we, therefore, hypothesized that oxidative damage by singlet oxygen to mitochondria initiates a secondary wave generation of ROS to amplify the damaging effects. Four hours after MG-2I and light exposure, we observed a significant increase in MitoSox signal (79.3% of cells exhibited increased superoxide generation) compared with MG-2I or light exposure alone (0.3%) (Fig. 3*A*), indicating stimulation of superoxide after singlet oxygen insult. Importantly, 5-min exposure to singlet oxygen was able to initiate a chronic flux of superoxide generation that lasted for at least 48 h (74.8% of cells at 24 h and 70.7% of cells at 48 h showed elevated superoxide), suggesting the occurrence of a continuous cycle of ROS generation in mitochondria. MitoTEMPO, a mitochondrial-targeted superoxide scavenger (26), was able to suppress MitoSox signal (36% reduction), further confirming the generation of superoxide in mitochondria (Fig. 3*B*).

To assess the potential sites of superoxide generation within the ETC, we used several inhibitors against specific ETC components. While both rotenone (Complex I inhibitor) and antimycin A (Complex III inhibitor) further enhanced superoxide generation by MG-2I and light treatment (*SI Appendix*, Fig. S3), 2-thenoyltrifluoroacetone (TTFA; Complex II inhibitor) had no effect. Since singlet oxygen did not immediately suppress the activity of these ETC complexes and because these inhibitor treatments increased rather than decreased superoxide production compared with previous reports (27, 28), our data suggested that a secondary wave of superoxide and hydrogen peroxide makes the ETC more prone to inhibitor-mediated ROS production.

Singlet oxygen, due to its high reactivity, does not diffuse far (∼20 nm) from its site of generation before it reacts with other moieties (29, 30). Superoxide anion generated in mitochondrial matrix, due to its negative charge, is not able to cross the mitochondrial inner membrane. However, superoxide is rapidly converted to hydrogen peroxide by MnSOD in mitochondria. Hydrogen peroxide is freely diffusible, causing oxidation in other cellular compartments beyond mitochondria. We monitored the cellular redox status of cysteine that can be readily oxidized by hydrogen peroxide using a ratiometric single-cell redox imaging method, in which free sulfhydryl groups are first labeled with thiol-reactive dye Alexa Fluor 647-maleimide followed by reduction of oxidized thiols (present and unreactive as disulfides during the initial labeling) by Tris(2-carboxyethyl)phosphine (TCEP) and subsequent labeling of these reduction-activated sulfhydryl groups with a second thiol-reactive dye Alexa Fluor 555-maleimide (31). As shown in Fig. 3 *C* and *D*, there was a significant increase in the ratio of fluorescence intensity of Alexa Fluor 555/647 (oxidized thiols/reduced thiols) in the nuclear region of the cells after MG-2I and light treatment, indicating the oxidation of nuclear protein after mitochondrial damage. A 24% increase in the fluorescence of a nuclear H_2_O_2_ sensor pHyper-nuc after treatment with MG-2I and light, compared with control, also supports the presence of H_2_O_2_ in the nucleus as a result of prolonged mitochondrial dysfunction (Fig. 3*E*).

### Mitochondrial Dysfunction Induces Nuclear DNA Replication Stress without Cell Death.

**Fig. 4.**
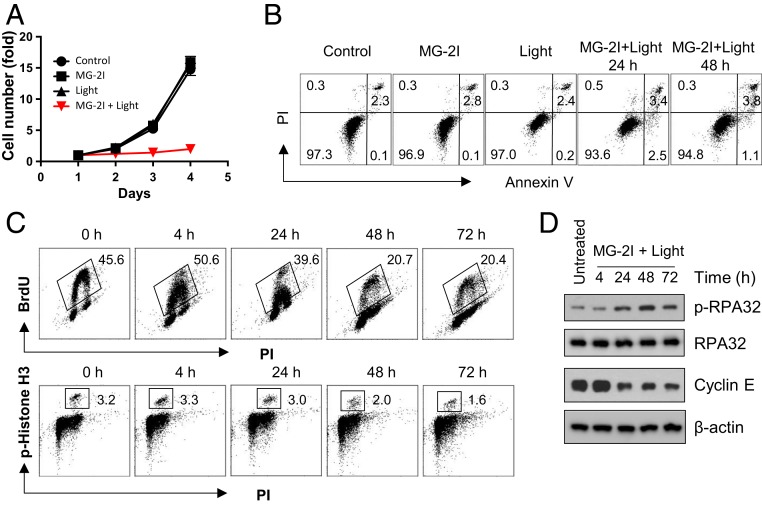
Mitochondrial dysfunction leads to nuclear DNA replication stress. (*A*) HEK293 Mito-FAP cells were treated with MG-2I (50 nM), light (5 min), or light (5 min) in the presence of MG-2I (50 nM). Cell proliferation was determined by a CyQuant assay at indicated time points. Data are represented as mean ± SD of 4 wells. A representative experiment is shown. (*B*) Apoptotic cell death in HEK293 Mito-FAP cells after treatment with MG-2I (50 nM), light (5 min), or light (5 min) in the presence of MG-2I (50 nM) was determined by Annexin V and PI staining at indicated time points. Numbers indicate the percentages of each population positive or negative with Annexin V or PI staining. (*C*) The effect of the exposure to light (5 min) in the presence of MG-2I (50 nM) on cell cycle progression was determined by BrdU incorporation (DNA replication) and phosphorylation of histone H3 (mitosis). Numbers indicate the percentages of cell population positive for incorporating BrdU or phosphorylated histone H3. (*D*) The phosphorylation of RPA32 and the protein level of Cyclin E after HEK293 Mito-FAP cells treated with light (5 min) in the presence of MG-2I (50 nM) were determined by western blot.

We next sought to determine the biological consequences of this secondary wave of ROS produced by the singlet oxygen-injured mitochondria. We observed a significantly reduced cell growth after treatment with MG-2I dye and light (50 nM MG-2I with light exposure for 5 min). Seventy-two hours after treatment, the cell number in the group treated with the combination of MG-2I and light was only doubled, which is around 7-fold lower than in control cells or cells treated with dye or light alone (Fig. 4*A*). To analyze if the reduced cell growth is due to enhanced cell death, we performed the Annexin V apoptosis assay. We did not detect a significant apoptosis at 24 or 48 h in cells treated with MG-2I and light (Fig. 4*B*), indicating a cell cycle delay due to dysfunctional mitochondria. We thus investigated cell cycle changes by monitoring S-phase bromodeoxyuridine (BrdU) incorporation and mitotic phosphorylation of histone H3 (Fig. 4*C*). Starting from 4 h after MG-2I and light treatment, we observed a decrease in the cellular intensity of BrdU signal (the amount of BrdU incorporated per cell) followed by a reduced number of cells incorporating BrdU over the next 24- to 72-h period (45.6% of cells are BrdU positive in control vs. 20.4% in cells 72 h posttreatment). There was no significant change in the number of mitotic cells as revealed by the unaltered number of cells positive for phosphorylated histone H3. We also observed a time-dependent increase in the phosphorylation of RPA32 at Ser4/Ser8, a marker of replication stress, and a decrease of Cyclin E, an S-phase cyclin, demonstrating nuclear DNA replication stress as a result of mitochondrial dysfunction (Fig. 4*D*).

### Mitochondrial Dysfunction Leads to Activation of the ATM Pathway in the Absence of Overall Nuclear DNA Strand Breaks.

**Fig. 5.**
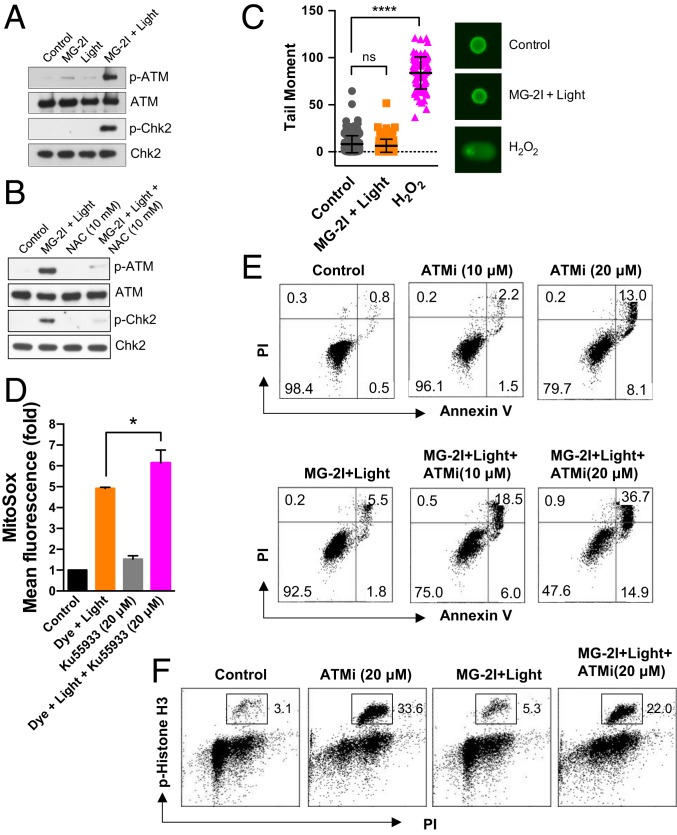
Activation of the ATM pathway in response to oxidative stress after MG-2I and light treatment. (*A*) At 24 h after MG-2I (50 nM) and light (5-min) treatment, phosphorylation of ATM and Chk2 was determined by western blot. (*B*) Cells were treated with NAC (10 mM) for 15 min before, during, and after exposure to MG-2I dye (50 nM) and light (5 min). At 24 h postexposure, the phosphorylation of ATM and Chk2 was determined by western blot. (*C*) DNA single-strand breaks were determined by alkali Comet assay 24 h after treatment with light (5 min) in the presence of MG-2I (50 nM). Treatment of 100 μM H_2_O_2_ for 20 min served as a positive control. Data are represented as mean ± SEM. Control (*n* = 235), MG-2I + Light (*n* = 263), and H_2_O_2_ (*n* = 91). ns, not significant. ****P < 0.0001. (*D*) Cells were treated with ATM kinase inhibitor KU-55933 alone, MG-2I (50 nM) plus light (5 min), or MG-2I plus light exposure in the presence of KU-55933. The generation of mitochondrial superoxide was determined 4 h after treatment by MitoSox. Data are represented as mean ± SEM. *P < 0.05. (*E*) Cells were treated in a manner as described in *D*, and the apoptosis was determined 72 h after MG-2I and light treatment. (*F*) The number of cells in mitosis was measured by the phosphorylation of histone H3 24 h after treatment.

Since a significant flux of hydrogen peroxide generated by mitochondria reached the nucleus, we sought to investigate whether DNA damage and repair signaling that may be involved in the cell cycle changes. After MG-2I and light exposure, we observed phosphorylation of ATM and Checkpoint kinase 2 (Chk2) (Fig. 5*A*), the signaling pathway that is typically activated by DNA double-strand breaks. Activation of ATM has also been known to be directly induced by hydrogen peroxide (32, 33). As shown in Fig. 5*B*, the antioxidant NAC suppressed the phosphorylation of both ATM and Chk2 after MG-2I and light treatment. However, the lack of ATM dimer formation after MG-2I and light treatment (*SI Appendix*, Fig. S4*A*) indicated that the activation of ATM is not due to direct oxidation of ATM protein and is probably a result of DNA double-strand breaks secondary to oxidative DNA damage. In order to confirm the generation of DNA damage, including double-strand breaks, after MG-2I and light treatment, we examined the phosphorylation of KRAB-associated protein-1 (KAP1), which is activated by DNA double-strand breaks but not oxidation (33). A small amount of KAP1 phosphorylation was detected after MG-2I and light exposure (*SI Appendix*, Fig. S4*B*), confirming the occurrence of nuclear DNA double-strand breaks, although to a limited extent. However, as revealed by an alkaline Comet assay, surprisingly, no significant overall nuclear DNA strand breaks (including both single- and double-strand breaks) were observed after MG-2I and light treatment compared with 20-min H_2_O_2_ (100 μM) treatment (Fig. 5*C*). The lack of detectable nuclear DNA strand breaks by the Comet assay is also consistent with our DNA damage assay by qPCR, which showed no reduction in the amplification of nuclear polymerase β gene (Fig. 2*F*). These results suggested the presence of a specific type of DNA strand break that activates an ATM response but is undetectable to the Comet assay.

To understand the role of ATM in cellular dysfunction induced by mitochondrial damage, we studied the effect of ATM inhibitor (ATMi) KU55933 on cell death and cell cycle changes after photosensitization. KU55933 further enhanced mitochondrial generation of superoxide (Fig. 5*D*) and synergistically induced apoptosis in cells treated with MG-2I and light (Fig. 5*E*) (e.g., 24.5% of Annexin V-positive cells were observed in cells treated with MG-2I + light+ 10 μM ATMi vs. 3.7% of Annexin V-positive cells were observed in cells treated with ATMi alone and 7.3% of Annexin V-positive cells were observed in cells treated with MG-2I and light). A protective ATM role on injured mitochondria after MG-2I and light treatment is consistent with the previous observation of mitochondrial dysfunction in ATM knockout cells (34). Furthermore, Fig. 5*F* revealed that 22% of cells undergo mitosis after treatment with MG-2I + light + ATMi. In contrast, the majority of cells treated with MG-2I + light showed S-phase delay (Fig. 4*C*), and only 5.3% of those cells undergo mitosis (Fig. 5*F*). Thus, the result in Fig. 5*F* indicated that the inhibition of ATM overrides replication stress-mediated S-phase delay after MG-2I and light treatment, forcing cells to progress into mitosis under replicative stress. The combination of enhanced mitochondrial superoxide generation and forced mitotic entry may underlie the mechanism of synergistic cell killing by the combination of ATM inhibition and FAP-bound MG-2I activation.

### Mitochondrial Dysfunction Leads to Telomere Damage.

**Fig. 6.**
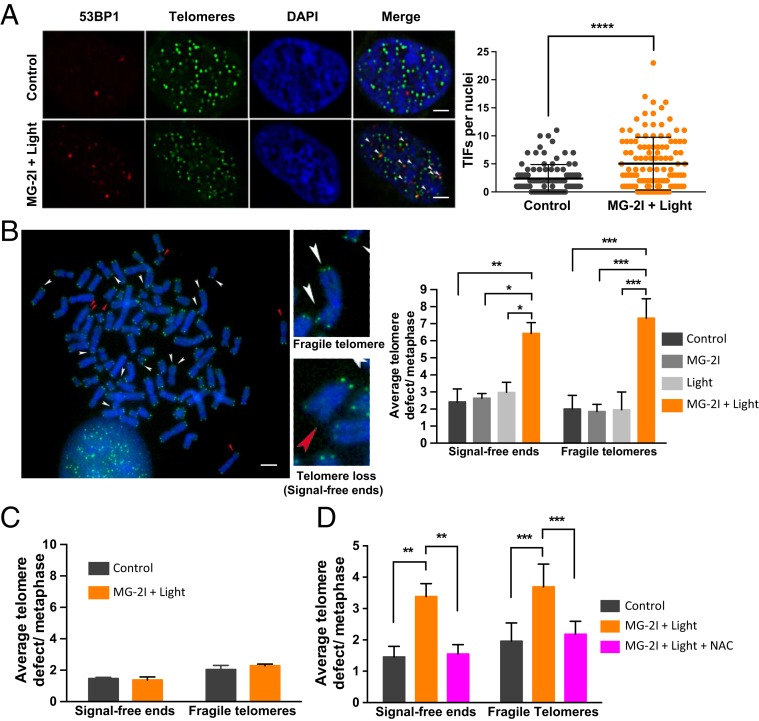
Mitochondrial dysfunction leads to telomere damage. (*A*) The recruitment of 53BP1 (red) at the telomeres (green) 48 h after MG-2I (50 nM) and light (5-min) treatment was analyzed by immunofluorescence. The number of 53BP1^+^/PNA^+^ TIFs was quantified. Data are represented as mean ± SD. ****P < 0.0001. (Scale bars: 2 μm.) (*B*) The number of HEK293 Mito-FAP cells with fragile telomere and telomere loss (signal-free ends) was evaluated by FISH 48 h after MG-2I (50 nM) and light (5-min) treatment. Data are represented as mean ± SD. (Scale bar: 2 μm.) (*C*) Telomere damage in HEK293 Mito-FAP ρ^0^ cells was analyzed as described in *B*. Data are represented as mean ± SD. (*D*) HEK293 Mito-FAP cells were pretreated with NAC (10 mM) for 15 min before, during and after light (5-min) exposure, and their telomere damage was then analyzed as in *B*. Data are represented as mean ± SD. At least 50 cells were counted. Representative results from 3 independent experiments are shown. *P < 0.05, **P < 0.01, ***P < 0.001.

Linn and coworkers (35) have shown that telomeric DNA sequences, TTAGGG, are 7-fold more likely to be damaged by hydrogen peroxide due to the propensity of iron to bind to these sequences and mediate Fenton chemistry. Considering the lack of an overall detectable increase in DNA strand breaks (Fig. 5*E*), in order to analyze whether DNA double-strand breaks occur at telomeres after mitochondrial dysfunction, driving the phosphorylation of KAP1 and ATM, we examined the localization of 53BP1, a DNA repair protein for double-strand breaks, which is known to be recruited to telomeres upon double-strand break formation (36). The number of TIFs was analyzed by the colocalization of 53BP1 and peptide nucleic acid (PNA) telomere probe; 53BP1^+^/PNA^+^ nuclear foci were defined as TIFs, and 53BP1^+^/PNA^−^ nuclear foci were defined as non-TIFs. We observed a significant 2-fold increase in the number of TIFs (2.2 in control vs. 4.8 in the MG-2I plus light-treated group), indicating the recruitment of 53BP1 to telomeres 48 h after MG-2I and light exposure in HEK293 Mito-FAP cells (Fig. 6*A*). In contrast, the number of non-TIFs (2 in control vs. 2.3 in the MG-2I plus light-treated group), which indicates 53BP1 foci that are not associated with telomeres (*SI Appendix*, Fig. S5*A*), remained unchanged. The increase in the TIFs indicated that DNA double-strand breaks exclusively occurred at telomeres, even in the absence of overall DNA strand breaks (Figs. 2*F* and 5*E*). Furthermore, there was a 2.6-fold increase in the percentage of cells consisting of >5 telomeric 53BP1 foci when treated with MG-2I and light (*SI Appendix*, Fig. S5*B*). Examination of telomeres 48 h after MG-2I and light treatment indicated a significant increase in fragile telomeres (2 in control vs. 7.3 in the MG-2I plus light-treated group) (shown by multimeric telomeric signals at a chromatid end in Fig. 6*B*) and telomere losses (2.4 in control vs. 6.4 in the MG-2I plus light-treated group) (shown by lack of telomeric signal at a chromatid end in Fig. 6*B*), which is consistent with telomeric DNA damage. Since the secondary wave of superoxide and hydrogen peroxide production is dependent on dysfunctional ETC, we reasoned that ETC-deficient ρ^0^ cells would not show telomeric DNA damage after MG-2I and light exposure; ρ^0^ cells are devoid of mtDNA and hence, unable to generate a significant flux of ROS from ETC (9); therefore, they should not be able to produce sufficient hydrogen peroxide after MG-2I and light treatment to cause telomere damage. As a result, we did not find any increase of telomere defects in HEK293 Mito-FAP ρ^0^ cells (Fig. 6*C*), indicating that the lack of ROS production renders cells resistant to telomere damage. Similarly, NAC was also able to effectively prevent damage at telomeres after MG-2I and light exposure (Fig. 6*D*). These results indicated that telomeres are especially sensitive to oxidative damage, leading to telomere defects as a direct result of mitochondrial injury.

## Discussion

**Fig. 7.**
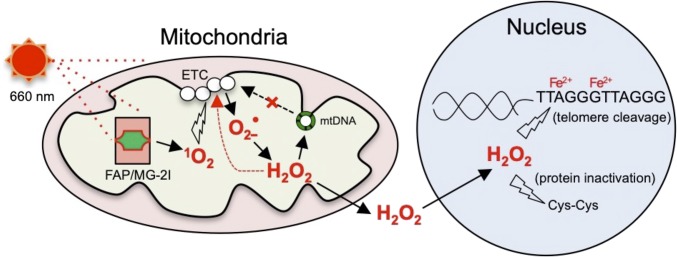
Working model of how mitochondrially generated hydrogen peroxide causes telomere damage. On 660-nm light exposure, the complex of Mito-FAP and MG-2I produces singlet oxygen. Singlet oxygen can induce oxidative damage to mitochondrial ETC, initiating a persistent secondary wave of superoxide and hydrogen peroxide generation. Hydrogen peroxide generated by mitochondria is able to damage mtDNA, which amplifies the damage to ETC. Hydrogen peroxide can further diffuse to the nucleus and is sufficient to cause nuclear protein oxidation and preferential telomere DNA damage but not overall nuclear DNA damage.

In this study, we have provided direct evidence that mitochondrial dysfunction induced by mitochondrial-targeted singlet oxygen is able to initiate a persistent secondary wave of superoxide and hydrogen peroxide generation. Importantly, hydrogen peroxide generated by mitochondria can diffuse to the nucleus and is sufficient to cause preferential telomere dysfunction but not overall nuclear DNA damage (Fig. 7).

Many environmental factors, such as heavy metals, sunlight, and pesticides, are known to cause mitochondrial dysfunction, ROS generation, and/or telomere damage, leading to pathological conditions (37–41). However, the relationship between mitochondria and telomere injury remained elusive, partly due to the inability of experimentally restricting damage exclusively to either compartment within a living cell. We have previously established a light-activated photosensitizer system that targets FAP to various cellular compartments combined with irradiation with light to precisely control the generation of singlet oxygen causing damage to those distinct compartments (18). In this study, by using Mito-FAP, we were able to initiate mitochondrial damage by generating singlet oxygen exclusively in mitochondria. The mitochondrial damage resulting from singlet oxygen generated in the mitochondria is probably due to initial direct oxidation of mitochondrial ETC proteins by singlet oxygen followed by the secondary generation of superoxide and hydrogen peroxide from dysfunctional mitochondrial respiration. Significantly, by confining the initial insult to the mitochondria to a short duration during which light was applied, we were able to follow the subsequent flux of distinct ROS and provide direct evidence that mitochondrial-generated hydrogen peroxide is able to travel to the nucleus and damage macromolecules inside the nucleus.

One of the advantages of the Mito-FAP system is the ability to fine tune the oxidative damage by adjusting the light exposure (*SI Appendix*, Fig. S2). With a condition that only suppresses cell growth without inducing significant cell death (Fig. 4), the Mito-FAP system allowed us to dissect the signaling events in the nucleus after mitochondrial dysfunction in a more physiologically relevant manner in contrast to many oxidative agents that often lead to gross nuclear DNA cleavage and cell death. While it has been suggested that oxidative stress causes telomere damage, such as telomere shortening and uncapping, it was not clear whether ROS generated by mitochondria under pathophysiological stress conditions is sufficient to directly cause telomere alterations (12). In our study, after an increase of mitochondrial ROS triggered by MG-2I and light treatment-induced singlet oxygen, we were also unable to detect gross nuclear DNA damage by either qPCR-based DNA damage assay or alkaline Comet assay for DNA single-strand breaks (Fig. 5*E*), which is consistent with previous reports (8, 9). However, targeted analysis of telomere lesions revealed telomeres as a preferential damage site for mitochondrial-generated ROS within the nuclear DNA. It is worth noting that DNA strand breaks at telomeres are not detectable by Comet. The absence of telomere damage in ρ^o^ cells after MG-2I and light treatment (Fig. 6*C*) and the protective effect of NAC (Fig. 6*D*) further demonstrate that ROS is essential in mitochondrial dysfunction-induced telomere damage. These results provide clear evidence that mitochondrial dysfunction directly leads to oxidative stress-mediated preferential telomere damage in the absence of general nuclear DNA damage.

The mechanism of the high susceptibility of telomere DNA to mitochondrial-generated oxidative stress has several potential causes. First, Linn and coworkers (42, 43) have shown that telomeric sequences are highly susceptible to hydrogen peroxide damage due to increased affinity for Fe^2+^ atoms that are bound to DNA to mediate Fenton chemistry. They reported that the preferential strand cleavage of DNA treated with hydrogen peroxide occurs at the nucleoside 5′ dG moieties in the sequence RGGG, a sequence found in telomere repeats (35). In fact, plasmids containing 81 telomeric repeats are 7-fold more sensitive to damage by hydrogen peroxide plus iron (35). Second, mitochondria are the major site for the biosynthesis of iron-sulfur (Fe/S) clusters, which are critical prosthetic groups for several key proteins involved in nuclear maintenance, such as DNA polymerases and helicases (44, 45). RTEL1, regulator of telomere elongation helicase 1, an Fe/S cluster-containing helicase, is an essential helicase for the control of telomere length and DNA repair on telomeres (46). When under oxidative stress, mitochondrial dysfunction can directly impair the biosynthesis of Fe/S clusters. In addition, the secondary ROS originated from mitochondria can cause potential Fe/S cluster conversion or complete cluster loss in proteins required for telomere maintenance.

The observation of 53BP1 recruitment to the TIFs 48 h after mitochondrial injury indicated direct DNA damage or uncapping at telomeres that are caused by mitochondrial-generated ROS. It has been reported that double-strand breaks at telomeres induced by a targeting Fok1-TRF1 system activate ATM kinase, which then leads to the accumulation of DNA damage and repair factors, such as 53BP1, to the break sites (36). Recruitment of 53BP1 in telomere DNA damage foci is known to increase chromatin mobility, promoting nonhomologous end joining DNA repair (36). Since we were not able to detect overall DNA strand breaks in nuclear DNA by the Comet assay and because phosphorylation of KAP1 is mediated by double-strand breaks and not oxidation (33), the weak but detectable phosphorylation of KAP1 observed in Fig. 5*D* is likely the direct consequence of double-strand breaks at telomeres. Furthermore, the cell cycle arrest that we observed after Mito-FAP/MG-2I and light exposure (Fig. 4) may be partly due to telomere damage, as activation of ATM kinase after telomere damage has been shown to lead to cell cycle arrest (47). Our results demonstrating that ATM kinase inhibitor overrode cell cycle arrest caused by Mito-FAP/MG-2I and light exposure, leading to cell death (Fig. 5*F*), are also consistent with the role of ATM in telomere damage and cell cycle alterations.

Both mitochondrial and telomere functions are critical for cellular senescence, apoptosis, aging, and cancer (48). Using mitochondrial-targeted chemoptogenetic tools, we established that disrupted mitochondrial function is sufficient to damage telomeres through ROS intermediates in the absence of gross nuclear DNA damage. Such mitochondria–telomere axes of damage could serve as an originating event (for example, in malignant transformation) by producing conditions that favor survival of cells that possess high levels of telomere repair and maintenance capacity, a common feature of oncogenic cells. Since progeroid transgenic mice with critically short telomeres have mitochondrial dysfunction and increased production of ROS (49), our observation also suggests a cross-talk between dysfunctional mitochondria and dysfunctional telomeres that may set up a continuous cycle of cellular events, further amplifying this reciprocal damage in the cell. The prolonged mitochondrial generation of ROS after Mito-FAP/MG-2I and light treatment in the absence of significant apoptosis may be the manifestation of the occurrence of such a chronic cycle of damage. Hydrogen peroxide may, therefore, serve as a direct mediator of mitochondrial retrograde signaling pathway, regulating telomere-related cellular function in response to mitochondrial functional alterations under oxidative stress. The mitochondrial oxidative damage, persistent generation of ROS, and the resulting telomere dysfunction after initial transient singlet oxygen exposure may underlie the molecular processes of cancer, photoaging, and other human pathologies.

## Materials and Methods

### Cell Culture.

HEK293 cells expressing Mito-FAP dL5** (Mito-FAP, FAP cloned downstream of mitochondrial targeting sequence of COXIV/COXVIII and fused with a fluorescent protein mCerulean3 for visualization) (18) were cultured in Dulbecco’s modified Eagle medium (DMEM) media supplemented with 10% heat-inactivated fetal calf serum (FCS) and 1% penicillin-streptomycin in 5% CO_2_ at 37 °C. The HEK293 Mito-FAP ρ^0^ cell line was established by culturing parental HEK293 Mito-FAP cells in DMEM supplemented with 10% heat-inactivated FCS, 1% penicillin-streptomycin, 1 mM sodium pyruvate, 50 μg/mL uridine, and 50 ng/mL ethidium bromide for at least 4 wk (50). pHyPer-nuc plasmids were obtained from Evrogen. Transfection was performed using FuGENE 6 (Roche Diagnostics) according to the manufacturer’s instructions.

### FAP Activation of MG-2I Dye.

HEK293 Mito-FAP cells were incubated at 37 °C for 15 min in phenol red free DMEM media containing MG-2I at a final concentration of 50 nM. Cells were then exposed to 660-nm (0.1-W/cm^2^) light for 5 min to stimulate the production of singlet oxygen. Phenol red free medium was then removed, and fresh growth medium was applied for all subsequent procedures.

### Stimulated Emission Depletion Microscopy (STED) Imaging.

HEK293 cells stably expressing a mitochondrial-directed FAP were plated in an uncoated Mattek glass-bottomed 35-mm culture dish (Mattek Corporation) and infected with an adenovirus coding for an mNEON-TOM20 construct. This protein is specifically localized to the mitochondrial outer membrane. Mito-FAP was imaged using MG-ester. Images were collected using a Leica 3× STED microscope using a white light laser tuned at 488 nm (mNEON) and 640 nm (FAP). Depletion was with the 775-nm line.

### Western Blot Analysis.

Western blot was performed as we previously described (51). Primary antibodies against ATM and β-actin were obtained from Sigma-Aldrich. Drp1 was from BD Biosciences. Phospho-Drp1 (Ser616), phospho-Chk2 (Thr68), and Chk2 were from Cell Signaling Technology. Phospho-ATM (S1981) was from Epitomics. Complex I subunit NDUFS3, Complex I subunit NDUFB6, and Complex IV subunit II were from abcam.

### Cell Proliferation and Cytotoxicity Assay.

Cell proliferation was determined using a CyQUANT Direct Cell Proliferation Assay kit (Invitrogen) according to the manufacturer’s instructions. An fluorescein isothiocyanate (FITC) Annexin V Apoptosis Detection Kit (BD PharMingen) was used to quantify apoptotic cells according to the manufacturer’s instructions.

### Cell Cycle Analysis.

Cell cycle analysis was performed as we previously described (52). At various time points after treatment with MG-2I alone, light alone, or the combination of MG-2I and light, S-phase cells were pulse labeled with 10 μM BrdU for 30 min at 37 °C. Cells were fixed in 70% ice-cold ethanol overnight at 4 °C. DNA was denatured in 2 N HCl containing 0.5% Triton X-100 and neutralized with 0.1 M Na_2_B_4_O_7_. Cells were then stained with FITC-labeled anti-BrdU antibody (BD Biosciences) followed by Alexa Fluor 647-conjugated phosphohistone H3 (Ser-10) antibody (Cell Signaling Technology). DNA content was determined by incubating cells with propidium iodide (PI) solution (phosphate-buffered saline [PBS] containing 50 μg/mL PI and 40 μg/mL RNase A) for 30 min at room temperature. Samples were then analyzed on Accuri C6 flow cytometer (BD Accuri Cytometers); 1 × 10^5^ events per sample were acquired to ensure adequate mean fluorescence levels.

### Extracellular Flux Analysis.

OCR and ECAR were measured using a Seahorse XF96 Extracellular Flux Analyzer (Seahorse Bioscience) essentially as previously described (53). After treatment, cells were seeded in XF96 cell culture plates at 8 × 10^4^ cells per well in the presence of Cell-Tak cell and tissue adhesive. Cells were then washed, and growth medium was replaced with bicarbonate-free medium. Thereafter, cells were incubated for another 60 min in a 37 °C incubator without CO_2_ followed by simultaneous OCR and ECAR measurements.

### Mitochondrial Membrane Potential and ROS Generation.

To measure mitochondrial-generated ROS or mitochondrial membrane potential, cells were incubated with 5 μM MitoSox (Invitrogen) or 2 μM JC-1 (Invitrogen), respectively, for 30 min at 37 °C. After washing with PBS, cells were collected and suspended in PBS containing 1% bovine serum albumin (BSA). The fluorescence intensity of MitoSox and JC-1 was analyzed using an Accuri C6 flow cytometer (BD Accuri Cytometers).

### qPCR-Based Mitochondrial and Nuclear DNA Damage Analysis.

Damage to mtDNA and nuclear DNA was assessed 24 h after Mito-FAP/MG-2I dye and light treatment as we described previously (54). Briefly, total cellular DNA was isolated with a DNeasy Blood & Tissue kit (Qiagen), and the concentration of isolated DNA was determined by PicoGreen (Invitrogen). qPCR reaction mixtures contained 15 ng of template DNA. Triplicate qPCR reactions for each treatment condition were performed with an LA PCR kit (Takara Bio Company) in a Biometra Professional standard thermocycler 96 (Biometra). The primer nucleotide sequences were as follows: for the 221-bp fragment of mitochondrial genome: 5′-CCCCACAAACCCCATTACTAAACCCA-3′ and 5′-TTTCATCATGCGGAGATGTTGGATGG-3′; for the 8.9-kb fragment of mitochondrial genome: 5′-TCTAAGCCTCCTTATTCGAGCCGA-3′ and 5′-TTTCATCATGCGGAGATGTTGGATGG-3′; and for the 12.2-kb human β-polymerase gene: 5′-CATGTCACCACTGGACTCTGCAC-3′ and 5′-CCTGGAGTAGGAACAAAAATTGCTG-3′. The amounts of the PCR products were quantified using PicoGreen to indicate relative amplification capability, which is dependent on the damage frequency of DNA template. Lesion frequency per DNA strand of treated samples was calculated based on the formula: λ = −lnF_T_/F_C_, where F_T_ is the fluorescence values of treated samples and F_C_ is the fluorescence values of control samples.

### Ratiometric Redox Immunocytochemistry.

The cellular oxidative status was measured by a ratiometric approach previously reported (31). After treatment, HEK293 Mito-FAP cells were fixed for 30 min in the dark in 4% paraformaldehyde, 1 mM *N*-ethylmaleimide (NEM), 2 μM Alexa Fluor 647-maleimide, and 0.02% Triton X-100 in PBS, resulting in the labeling of the sulfhydryl group with Alexa Fluor 647. Cells were washed 3 times for 5 min in PBS to remove excess dye and then, incubated for 30 min in 5 mM TCEP in PBS to reduce disulfide bonds in proteins. Reduced disulfide bonds were then labeled with Alexa Fluor 555 by incubating cells for 30 min in 1 mM NEM and 2 μL Alexa 555-maleimide. After 3 additional washes in PBS for 5 min, cells were examined and photographed by a laser-scanning confocal microscope, Olympus FLUOVIEW FV-1000, with a PlanApo N 60× oil immersion objective and numerical aperture (NA) = 1.42 (Olympus). Ratiometric analysis on the fluorescence intensity of Alexa Fluor 555/647 SS/SH (disulfide/thiol) was performed using NIS-Elements software (Nikon).

### Alkaline Comet Assay.

The single-cell gel electrophoresis or Comet assay was performed with a CometAssay kit (Trevigen). Twenty-four hours after treatment with MG-2I alone, light alone, or the combination of MG-2I and light, cells were collected by trypsinization and centrifugation. Cells were then washed once in ice-cold PBS and suspended at 100,000 cells per 1 mL in ice-cold PBS. Cells were combined with molten low melting agarose (LMAgarose) (at 37 °C) at a ratio of 1:10 (vol/vol) and immediately spread onto a 20-well CometSlide. Slides were placed flat at 4 °C in the dark for 10 min to allow gelling. Slides were immersed in 4 °C lysis solution overnight. After draining excess lysis buffer, slides were gently immersed in freshly prepared alkaline unwinding solution, pH > 13 (200 mM NaOH, 1 mM ethylenediaminetetraacetic acid [EDTA]), for 20 min at room temperature. Electrophoresis was performed at 21 V for 30 min in alkaline electrophoresis solution pH > 13 (200 mM NaOH, 1 mM EDTA) with the CometAssay ES system. After electrophoresis, slides were immersed twice in distilled water (dH_2_O) for 5 min each followed by 70% ethanol for 5 min at room temperature. After drying overnight at room temperature, slides were stained with SYBR Gold for 30 min at room temperature. Images were taken with an Olympus BX51 microscope and analyzed by Trevigen’s Comet Analysis Software. At least 200 cells from each treatment condition were scored.

### Electron Transport Chain Complex Activity Assay.

The activity of citrate synthase was assessed by measuring the conversion of oxaloacetate with acetyl coenzyme A (CoA) to citrate + CoA as previously described (55). The production of CoA was coupled to its reaction with dithionitrobenzoic acid (DTNB), and the product of this reaction (DTNB-CoA) formed a colored compound with formation that was kinetically monitored spectrophotometrically at 412 nm. Lysed cells (10 to 30 μg) were equilibrated at 37 °C in a reaction mix containing 400 μM DTNB, 200 μM acetyl-CoA, 100 mM Tris, pH 8.0, and 0.1% Triton X-100. Reactions were initiated by the addition of 200 μM oxaloacetate. The increase in absorbance at 412 nm (10 min) and the rate were expressed as picomoles per minute per milligram of protein based on the extinction coefficient of 13,600 (moles per minute per liter) for DTNB-CoA.

The activities of the 4 ETC complexes were performed essentially as described previously (55). Complex I converts NADH to NAD+ using ubiquinone as a substrate. Complex I activity was measured by adding exogenous NADH and ubiquinone to the cells as substrates and monitoring the rotenone (10 µM)-sensitive decrease in the absorbance of NADH at 340 nm. Lysed cells were equilibrated at 37 °C in a reaction mixture containing 25 mM KPO_4_, pH 7.2, 10 mM MgCl_2_, 2.5 mg/mL BSA, 1 mM KCN, and 0.1 mM NADH. Reactions were initiated by the addition of decylubiquinone (50 μM), and the decrease in absorbance at 340 nm was monitored (10 min), after which rotenone (10 μM) and the reaction were monitored for an additional 10 min. The rotenone-sensitive rate was expressed as picomoles per minute per milligram of protein based on the extinction coefficient of 6,180 (moles per minute liter) for NADH.

Complex II activity was measured using succinate and ubiquinone as substrates, coupling the (a) Complex II-catalyzed transfer of an electron from succinate to ubiquinone and the (b) ubiquinol reduction of the dye 2,6-dichlorophenolindophenol (DCPIP), which is monitored at 600 nm. Lysed cells were equilibrated at 37 °C in a reaction mixture containing 50 mM KPO_4_, pH 7.4, 20 mM succinate, 0.1 mM EDTA, 1 mM KCN, 10 μM rotenone, and 0.12 mM DCPIP. Reactions were initiated by 50 μM decylubiquinone, and the decrease in absorbance at 600 nm was monitored for 10 min, after which TTFA was added to a final concentration of 1 mM and the reaction was monitored for an additional 10 min. The TTFA-sensitive rate was expressed as picomoles per minute per milligram of protein based on the extinction coefficient of 21,000 (moles per minute per liter) for DCPIP.

Complex III activity was measured using oxidized cytochrome *c* and ubiquinol as substrates and monitoring the reduction of cytochrome *c* at 550 nm. Lysed cells were added to a reaction containing 50 mM KPO_4_, pH 7.4, 1 mM EDTA, 5 mM MgCl_2_, 2 mM KCN, 10 nM rotenone, and 15 μM oxidized cytochrome *c*. Reactions were initiated by the addition of 15 μM ubiquinol. The increase in absorbance at 550 nm was monitored for 3 min. The first order rate constant (k) for the oxidation of cytochrome *c* was calculated and activity was expressed as k/min/mg. All complex activities are shown as a percentage of the control condition.

Complex IV activity was measured using reduced cytochrome *c* as the substrate and monitoring the oxidation of cytochrome *c* at 550 nm. Lysed cells were equilibrated to 30 °C in 10 mM KPO_4_, pH 7.0, and the reaction was initiated by the addition of 50 μM reduced cytochrome *c*. The decrease in absorbance at 550 nm was monitored for 3 min. The first order rate constant (k) for the oxidation of cytochrome *c* was calculated and activity was expressed as k/min/mg. All complex activities are shown as a percentage of the control condition.

### Mitochondrial Morphology Analysis.

At 4 and 24 h after MG-2I and light treatment, cells were fixed with 2% paraformaldehyde in PBS for 15 min at 37 °C. After permeabilization and blocking, a TOMM20 antibody (Santa Cruz Biotechnology) with an Alexa 555-conjugated secondary antibody was used to label and visualize the mitochondria. A Leica TCS SP8 STED 3× imaging system was used to obtain the superresolution images. A pulsed white light laser was used to excite at 554 nm, and emission was collected from 561 to 650 nm through Leica’s 100× 1.4 N.A. STED objective. Images are digitally zoomed to achieve superresolution Nyquist sampling at 19 nm per pixel, and the *z* stacks were collected at 100-nm steps. All images were deconvolved using Huygens Professional software (SVI). Three-dimensional mitochondrial surfaces were then reconstructed from *z* stacks, and mitochondrial connectivity was quantified using Imaris software (Bitplane).

### ATM Dimer Formation Analysis.

The formation of ATM dimer after MG-2I and light treatment was detected as described previously (56). Briefly, HEK293 Mito-FAP cells were harvested, washed with PBS, and suspended in 1 mL PBS containing 100 mM NEM. Cell suspension was incubated on ice for 30 min. Cells were then lysed in Nonidet P-40 lysis buffer. Sodium dodecyl sulfate (SDS) was added at a final concentration of 1%. Lysate was centrifuged at 14,000 rpm for 10 min at 4 °C. Supernatant was mixed with Laemmli buffer in the absence of BME or dithiothreitol (DTT) and without boiling. The formation of ATM dimer was then detected by western blotting.

### Metaphase Spreads and Telomere Fluorescence in Situ Hybridization (FISH) Analysis.

Cells were plated at a density of 4 × 10^5^ and 5 × 10^5^ per 100-mm dish for the control and treatment groups, respectively; 48 h after MG-2I and light treatment, cells were treated with 0.05 μg/ml colcemid for 2 h. Cells were then harvested and incubated with 75 mM KCl at 37 °C for 13 min followed by fixation with methanol:glacial acetic acid (3:1). Cells were either stored at −20 °C overnight or processed further. Cells were washed with the fixative again and dropped onto water-coated glass microscope slides to spread metaphase chromosomes. Slides were left to dry overnight at room temperature protected from light. The next day, the slides were fixed with 4% paraformaldehyde, washed with PBS, and treated with 0.25 mg/mL RNase A and 1 mg/mL Pepsin (in 0.01 N HCl) for 15 min each at 37 °C. Fixation and washing were repeated. Slides were dehydrated in 70, 90, and 100% ethanol, each for 5 min, and left to dry for at least 3 h at room temperature in the dark; 0.1 μM PNA probe [PNA Bio; F1004; (CCCTAA)_3_-Alexa 488] was diluted in hybridization solution (70% Di Formamide, 1× maleic acid, 10 mM Tris, pH 7.5, 1× MgCl_2_) and incubated at 95 °C for 3 to 5 min. Samples were hybridized for 10 min at 95 °C and stored overnight at 4 °C in a humid chamber box protected from light. The next day, the coverslips were removed, and slides were washed twice in hybridization wash buffer (70% Di Formamide, 10 mM Tris, pH 7.5, in dH_2_O) for 15 min followed by three 5-min washes (0.1 M Tris, pH 7.5, 150 mM NaCl, 0.08% Tween20 in dH_2_O). DAPI was added to the third wash. Finally, slides were washed twice in dH_2_O and dehydrated in 70, 90, and 100% ethanol, each for 5 min, before mounting with Prolong Diamond Anti-Fade (Molecular Probes). Images were acquired on the Nikon Ti inverted fluorescence microscope using *z* stacks of 0.2-μm thickness. Images were deconvolved and analyzed on NIS Elements Advanced Research software.

### Analysis of 53BP1 Foci at Telomeres.

HEK293 Mito-FAP cells were plated on coverslips; 48 h after MG-2I (50 nM) and light (5-min) treatment, cells were fixed with 4% paraformaldehyde for 10 min. After washing with PBS 3 times, cells were permeabilized with 0.2% Triton X-100 and blocked for 1 h at room temperature (10% goat serum, 1% BSA in PBS). The 53BP1 antibody (1:500; Santa Cruz Biotechnology) was added to the cells and incubated overnight at 4 °C. The next day, cells were washed 3 times with PBS and incubated with the secondary antibody (1:1,000; Cell Signaling Technology) for 1 h at room temperature. After washing 3 times with PBS, cells were fixed again with 4% paraformaldehyde for 10 min. Cells were washed again in PBS and dehydrated in 70, 90, and 100% ethanol for 5 min each; 0.1 μM PNA probe [PNA Bio; F1004; (CCCTAA)_3_-Alexa 488] was diluted in hybridization solution (70% Di Formamide, 1× maleic acid, 10 mM Tris, pH 7.5, 1× MgCl_2_) and incubated at 95 °C for 3 to 5 min. After letting the coverslips dry for 2 min, cells were hybridized for 10 min at 80 °C and incubated at room temperature for 2 h in a humid chamber box protected from light. Next, cells were washed twice in hybridization wash buffer (70% formamide, 10 mM Tris HCl, pH 7.5) for 15 min each. After 3 washes in PBS, cells were incubated with DAPI (1:10,000) for 10 min at room temperature. Cells were rinsed once with PBS and dH_2_O before being mounted on slides with Prolong Diamond Anti-Fade (Molecular Probes).

Images were acquired on a Nikon Ti inverted fluorescence microscope with a *z* stack of 0.2 μm. Images were deconvoluted and analyzed using NIS Elements Advance research software. The nuclei were labeled using the region of interest tool. Using the measurement tab, a separate binary layer was created for the 53BP1 foci and the telomere foci. The intersection tool was then used to create a third binary layer that identified the 53BP1 foci overlapping the telomere foci. The intensity threshold for each channel was kept the same for all samples. The foci counts were exported to Excel for analysis.

### Quantification and Statistical Analysis.

Data were expressed as mean ± SD. A Student’s *t* test was used for the comparisons between 2 groups. ANOVA was used to make comparisons between multiple groups. *P* < 0.05 was considered statistically significant.

## Supplementary Material

Supplementary File
