# Supplementary material for: Chemoptogenetic damage to mitochondria causes rapid telomere dysfunction
Source: Proc Natl Acad Sci U S A. 2019 Aug 26;116(37):18435–44. doi: 10.1073/pnas.1910574116 (PMC6744920; doi:10.1073/pnas.1910574116)
Supplement: Supplementary File [file pnas.1910574116.sapp.pdf]

PNAS
www.pnas.org

Supplementary Information for

Chemoptogenetic damage to mitochondria causes rapid telomere dysfunction

Wei Qian,[1] Namrata Kumar,[2] Vera Roginskaya,[1] Elise Fouquerel,[3] Patricia L. Opresko,[3] Sruti Shiva,[4] Simon C. Watkins,[5] Dmytro Kolodieznyi,[6] Marcel P. Bruchez,[6] Bennett Van Houten[1,*]

[1]Department of Pharmacology and Chemical Biology, University of Pittsburgh School of Medicine and UPMC Hillman Cancer Center, Pittsburgh, PA 15213, USA

[2]Department of Microbiology and Molecular Genetics, School of Medicine, University of Pittsburgh, Pittsburgh, PA 15213, USA

[3]Department of Environmental and Occupational Health, University of Pittsburgh Graduate School of Public Health and UPMC Hillman Cancer Center, Pittsburgh, PA 15261, USA

[4]Department of Pharmacology and Chemical Biology, Vascular Medicine Institute, University of Pittsburgh School of Medicine, Pittsburgh, PA 15261, USA

[5]Department of Cell Biology and Physiology, Center for Biological Imaging, University of Pittsburgh, Pittsburgh, PA 15261, USA

[6]Department of Chemistry, Department of Biological Sciences, and Molecular Biosensors and Imaging Center, Carnegie Mellon University, Pittsburgh, PA 15213, USA

*Corresponding Author

Corresponding author: Bennett Van Houten, PhD
Email: vanhoutenb@upmc.edu

This PDF file includes:

Figs. S1 to S5

www.pnas.org/cgi/doi/10.1073/pnas.1910574116

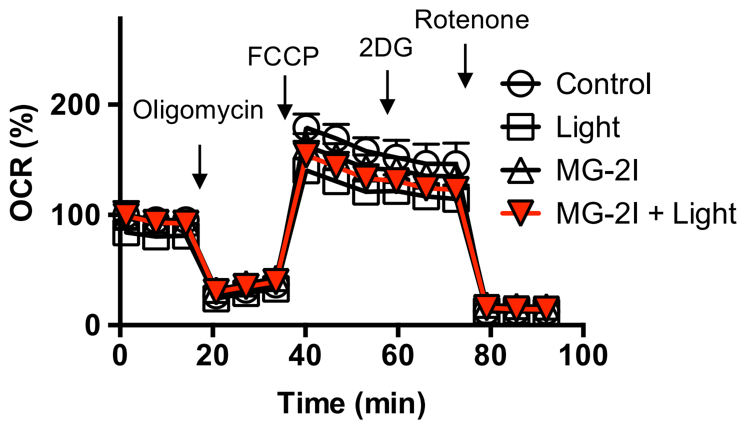

Supplemental Figure 1. HEK293 parental cells that do not express Mito-FAP show a lack of response to MG-2I and light treatment.
HEK293 parental cells were treated with MG-2I dye (50 nM) alone, light exposure (660nm, 5 min) alone, or light exposure (660nm, 5 min) in the presence of 50 nM MG-2I. Mitochondrial OCR was assessed by a Seahorse Extracellular Flux Analyzer 4 h after treatment. Data represents mean+/-SD of at least 6 wells.

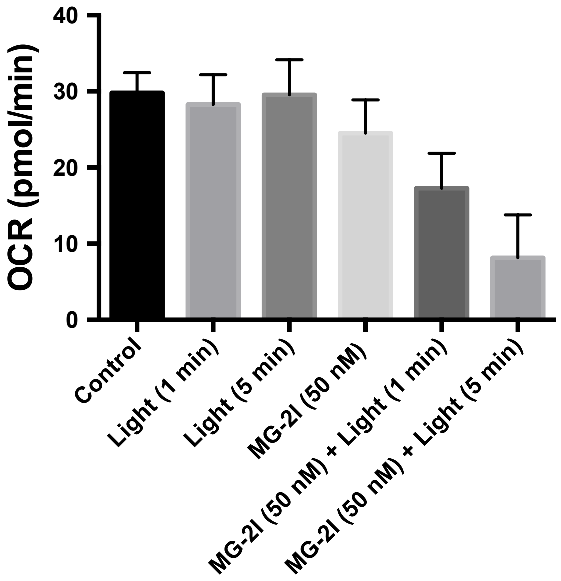

Supplemental Figure 2. The decrease of Oxygen Consumption Rate (OCR) is dependent on the time of light exposure.
HEK293 Mito-FAP cells were treated with MG-2I dye (50 nM) alone, light exposure (660nm, 1 min or 5 min) alone, or light exposure (660nm, 1 min or 5 min) in the presence of 50 nM MG-2I. Mitochondrial OCR was assessed by a Seahorse Extracellular Flux Analyzer 4 h after treatment. Data represents mean+/-SD of at least 6 wells.

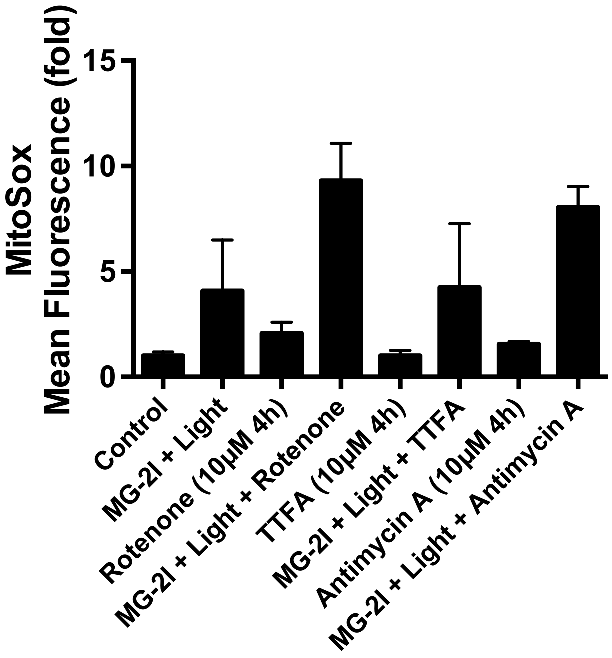

Supplemental Figure 3. Inhibitors of mitochondrial ETC complex I and III further enhanced mitochondrial superoxide generation following MG-2I and light exposure.
Complex I inhibitor rotenone, complex II inhibitor TTFA, and complex III inhibitor antimycin A were added 30 min before light exposure. Mitochondrial generation of superoxide was determined by MitoSox using flow cytometry, 4 h after treatment of HEK293 Mito-FAP cells with MG-2I (50 nM) and light (5 min). Data represent mean+/-SEM of three independent experiments.

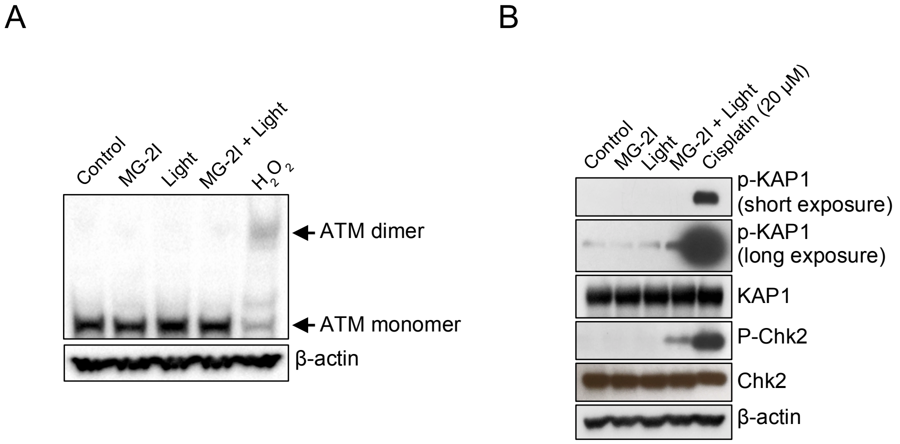

Supplemental Figure 4. ATM activation is independent of oxidative dimer formation.
A) HEK293 Mito-FAP cells were treated with MG-2I dye (50 nM) alone, light exposure (660nm, 5 min) alone, or light exposure (660nm, 5 min) in the presence of 50 nM MG-2I. 24 h after treatment, the formation of ATM dimer was examined by western blot, and H_2O_2 (100 μM, 30 min) was used as a positive control. B) The phosphorylation of KAP1 24 h after MG-2I (50 nM) and light (5 min) treatment, with cisplatin as a positive control, was determined by western blot.

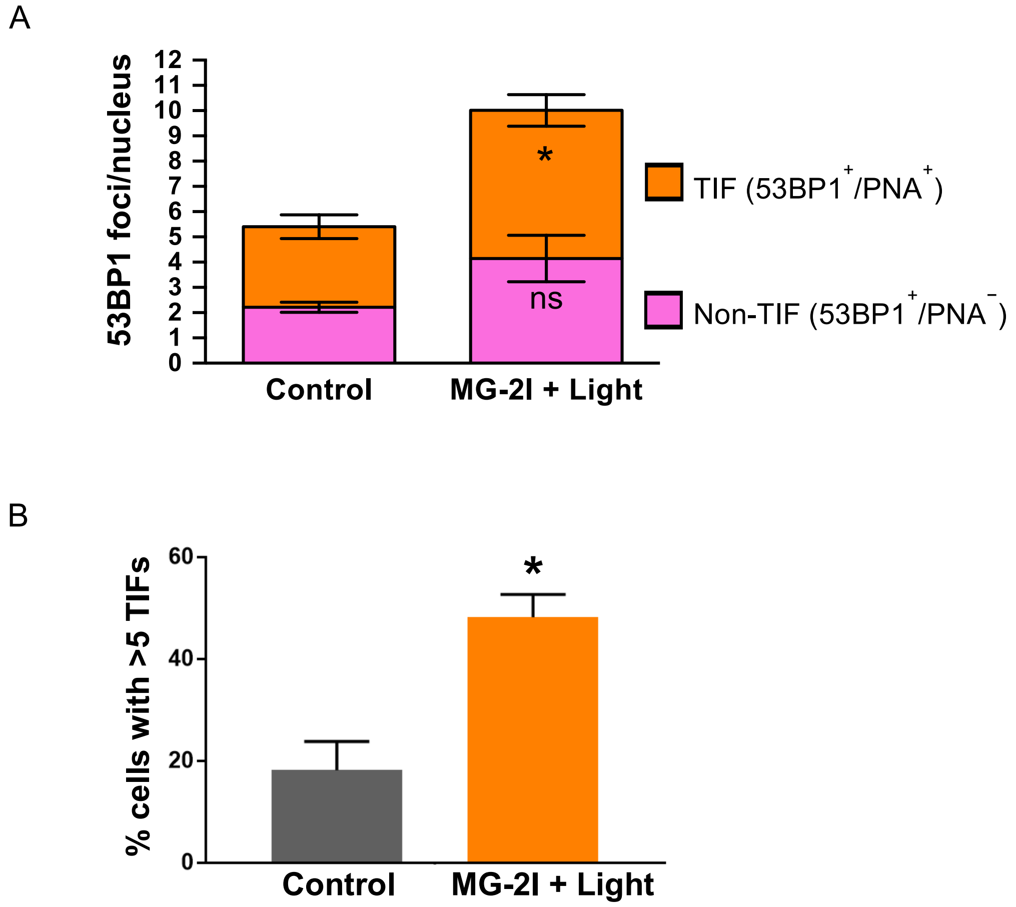

Supplemental Figure 5. Mitochondrial dysfunction induced by MG-2I and light treatment leads to 53BP1 foci formation primarily on telomeres.

A) HEK293 Mito-FAP cells were treated with MG-2I dye (50 nM) alone, light exposure (660nm, 5 min) alone, or light exposure (660nm, 5 min) in the presence of 50 nM MG-2I. The recruitment of 53BP1 to the telomeres, 48 h after treatment was analyzed by immunofluorescence. TIF was defined as $53BP1^{+}/PNA^{+}$ nuclear foci, and non-TIF was defined as $53BP1^{+}/PNA^{-}$ nuclear foci. B) The number of cells containing more than 5 TIFs were quantified after MG-2I and Light treatment as described in panel (A). Data represent mean+/-SEM of three independent experiments. *p<0.05. ns, not significant.
